# Epidemiologic Impact of Prioritizing Long-Acting Injectable Cabotegravir to Men Who Have Sex With Men With Low Pre-Exposure Prophylaxis Use Adherence in the Netherlands: A Mathematical Modeling Study

**DOI:** 10.1097/QAI.0000000000003882

**Published:** 2026-05-25

**Authors:** Haoyi Wang, Kai J. Jonas, David van de Vijver

**Affiliations:** a Department of Work and Social Psychology, Maastricht University, Maastricht, the Netherlands; and; b Viroscience Department, Erasmus Medical Centre, Rotterdam, the Netherlands.

**Keywords:** HIV, pre-exposure prophylaxis, long-acting PrEP, injectable PrEP, cabotegravir, men who have sex with men, Europe

## Abstract

**Background:**

Long-acting injectable cabotegravir (CAB-LA) as pre-exposure prophylaxis (PrEP), given once every 8 weeks, has been found to have superior efficacy over oral PrEP among men who have sex with men (MSM). We compared the epidemiologic impact of expanding CAB-LA to current oral PrEP users with different levels of adherence, and non-PrEP users among MSM in the Netherlands.

**Methods:**

An MSM HIV transmission model was calibrated to the Dutch MSM HIV epidemic. The real-world effectiveness of daily oral-PrEP was assumed to decrease by users' adherence levels: High (defined as >75% of the days covered, 93% effectiveness), medium (50%–74% covered, 69%), and low (<50% covered, 18%), respectively. CAB-LA effectiveness was assumed as 91%. CAB-LA was targeted to current oral-PrEP users by their use adherence and non-PrEP-using MSM in 2025. The epidemiologic impact of CAB-LA targeting strategies was evaluated for 5, 15, and 25 years.

**Results:**

Assuming 100% coverage, providing CAB-LA to MSM with low oral PrEP adherence would avert 862 (19.7%) new HIV infections among all MSM for 25 years. Expanding CAB-LA to MSM with medium/high oral PrEP adherence and non-PrEP users did not show a substantial epidemiologic impact. Moreover, our model suggested that the epidemiologic impact would decrease with a lower real-world CAB-LA effectiveness.

**Conclusions:**

Our model showed that CAB-LA can be an important addition to ending the HIV epidemic by 2030. To optimize the full potential of CAB-LA at the beginning stage of CAB-LA introduction, we suggest prioritizing the transition of current oral PrEP users with low adherence to CAB-LA to reduce HIV transmissions more efficiently.

## Abstract 2

Supplemental Digital Content is Available in the Text.

## INTRODUCTION

Oral tenofovir disoproxil fumarate/emtricitabine (TDF/FTC) as HIV pre-exposure prophylaxis (oral PrEP) is highly effective in preventing HIV infections.^1^ In the Netherlands, a resource-rich context with a declining HIV epidemic,^2^ oral PrEP has been formally introduced and implemented through a national pilot program since 2019 to support ending the HIV epidemic as a global health threat by 2030.^2^ This national PrEP program is primarily targeting men who have sex with men (MSM) with an increased vulnerability to HIV.^2,3^ However, HIV seroconversions still occurred along the PrEP care continuum in the Netherlands,^2,4^ partially because of users' suboptimal adherence.^5,6^

This corroborates concerns about oral PrEP users' suboptimal adherence,^1,7^ as being the major determinant of decreased effectiveness of the oral regimens.^1,6^ To overcome the challenges of suboptimal adherence and address the concerns associated with the oral regimens for non-PrEP users, including both PrEP-naïve individuals and those who have discontinued using oral PrEP, long-acting injectable cabotegravir (CAB-LA) has been proposed to reach a higher PrEP coverage and better prevention.^8,9^ Compared with oral PrEP, CAB-LA offers superior efficacy by alleviating the medication adherence challenges, because it only needs to be administered once every 8 weeks.^10^ Its official availability in Europe is expected soon, given that CAB-LA recently received its marketing approval from the European Medicines Agency.^11^ Hence, a coexistence of multiple PrEP modalities for HIV prevention would result.

In Europe, there are only 2 studies that have investigated the interest and preference for long-acting PrEP among MSM.^12,13^ Of these 2 studies, 1 study reported that long-acting injectable PrEP would capture the interest of 84% of MSM, with 68% of MSM currently using oral PrEP expressing a preference for long-acting injectable PrEP in the Netherlands.^12^ Although this evidence presents an important foundation for advocating the introduction of CAB-LA, it falls short in projecting the potential extent to which the utilization of CAB-LA, alongside the available oral PrEP, can further reduce the HIV epidemic. Therefore, it is crucial to further investigate the epidemiologic impact of how the introduction of CAB-LA can best be used alongside the use of oral PrEP.

However, to date, most existing studies investigating the potential epidemiologic impact of CAB-LA have exclusively concentrated on scenarios involving CAB-LA alone, overlooking the simultaneous presence of both oral regimens and CAB-LA.^14–17^ One exception, which aimed for model comparison with Dutch evidence, indicated that an extensive CAB-LA expansion to MSM could prove to be effective and efficient only within a high HIV incidence context such as Atlanta, but might not hold within a declining HIV epidemic characterized by low HIV incidence, as seen in the Netherlands.^18^ Moreover, this study also indicated that prioritizing CAB-LA for MSM with suboptimal adherence to oral PrEP could yield superior efficiency. However, this insight was derived solely from the Atlanta model, lacking corroborating evidence from the Netherlands model.^18^ This limited focus on CAB-LA prioritization emphasizes the need for a more comprehensive view of how to integrate it with oral PrEP. This is especially important in settings where the benefits of extensive CAB-LA expansion are relatively inefficient.^18^ Therefore, we must develop a strategy that leverages the public health potential of CAB-LA during its initial introduction.

Taken together, this study aims to assess which CAB-LA prioritizing strategy at the beginning stage of CAB-LA introduction would be the most effective and efficient for a declining HIV epidemic setting like the one in the Netherlands. For this purpose, this study modeled the epidemiologic impact of expanding CAB-LA to current non-PrEP users and MSM with different oral PrEP adherence. More importantly, this study modeled whether current oral PrEP using MSM with low adherence would benefit the most from using CAB-LA.

## METHODS

### Study Setting and Population

In the Netherlands, the HIV epidemic is well described through a national HIV database, which contains anonymized demographic and clinical data of >97% of people living with HIV in care.^2,19,20^ For this study, we adapted an existing validated compartmental deterministic mathematical model of HIV transmission among MSM.^21^ We further developed this model to be stratified by the current oral PrEP use status and adherence among MSM to represent the HIV epidemic among MSM aged 15 years and older in the Netherlands.

### Model Assumptions and Calibration

**FIGURE 1.**
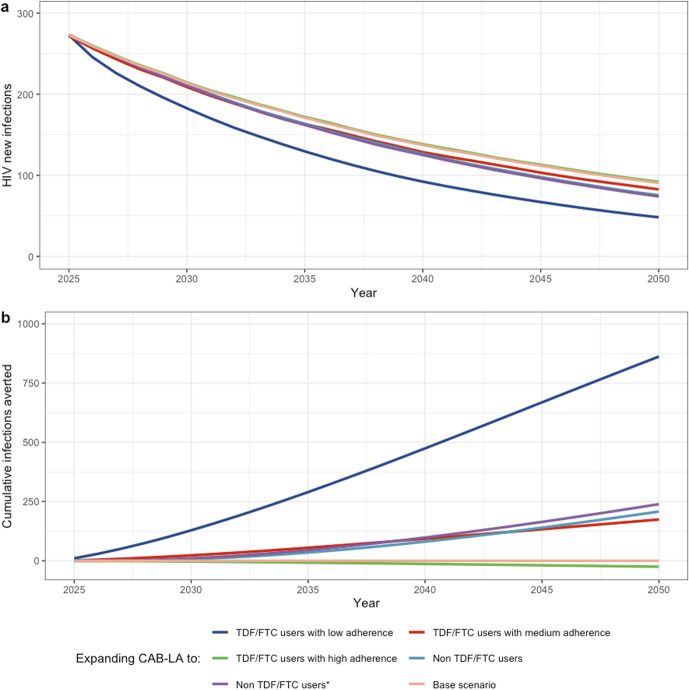
Projected population-level (A) MSM HIV new infections and (B) cumulative infections averted by simulated CAB-LA expansion scenarios for 25 years (2025–2050). For scenarios of expanding CAB-LA to currently oral PrEP users, a 100% oral PrEP to CAB-LA switching was assumed. For scenarios of expanding CAB-LA to currently nonoral PrEP users, a ratio of rates of CAB-LA/oral PrEP initiation =1 was assumed. *Indicates scenario assuming current nonoral PrEP users who initiate CAB-LA would adapt their sexual behaviors to be similar to current PrEP users. More details per scenario are given in the Supplemental Digital Content, http://links.lww.com/QAI/C679. Lines show median across 178 fits.

The mathematical model was seeded in 1981, stratifying HIV progression into the acute stage, 3 chronic stages (CD4>500 cells/µl, CD4 count 350–500 cells/µl, and CD4 count 200–349 cells/µl) and 1 AIDS stage (CD4 <200 cells/µl). The schematic representation of the model and the equations are in Figure 1, Supplemental Digital Content, http://links.lww.com/QAI/C679. Each stage of infection has a different duration and infectivity (Table 1). We assumed that MSM with HIV who were linked to treatment are virally suppressed and cannot transmit HIV to others.^2,22^

**TABLE 1.** Key Model Parameters of Oral PrEP and CAB-LA for HIV Prevention in the Netherlands

Model Parameters	Estimate or range*/IQR	References
Duration of disease stages		
Acute stage	10–16 wk	23
CD4^+^ T-cell count 350–500 cells/µL	2.9–3.1 yrs	24
CD4^+^ T-cell count 200–349 cells/µL	3.6–3.9 yrs	24
CD4^+^ T-cell count < 200 cells/µL	13–25 mo	24
Infectivity per partnership transmissibility per year		
Acute stage	0.030–0·61	25; Model calibration
Chronic stage	0.027–0·21	25; Model calibration
AIDS stage	0.008–0·27	25; Model calibration
Proportion MSM using TDF/FTC with different adherence†		
High	36% (25–45 range)	1; Model calibration, the sum of the 3 groups was equal to 100%
Medium	19% (10–30 range)	
Low	45% (25–62 range)	
Annual number of CAI with nonsteady partners by PrEP use status		
PrEP users	8.7	26
Non-PrEP users	3.0	
Mortality rates per year		
Population	0.0155	27
Chronic HIV stage	0.114	27
AIDS stage	0.648	27
On treatment	0.0184	27
PrEP regimen effectiveness		
Oral PrEP use with high adherence	93%	1
Oral PrEP use with medium adherence	69%	
Oral PrEP use with low adherence	18%	
CAB-LA	91%	10,14
PrEP regimens discontinuation rate		
TDF/FTC	0.71 (0.51–0.83 IQR)	Model calibration
CAB-LA	0.168	10,14

CAB-LA, long-injectable cabotegravir; IQR, interquartile range; MSM, men who have sex with men; PrEP, pre-exposure prophylaxis.

* All ranges were uniformly distributed.

† Definition of adherence followed the definition of Jourdain et al: high=>75%, median = 50%–75%, and low=<50% of oral PrEP use during follow-up (adherence), assuming a daily use scenario.

**FIGURE 2.**
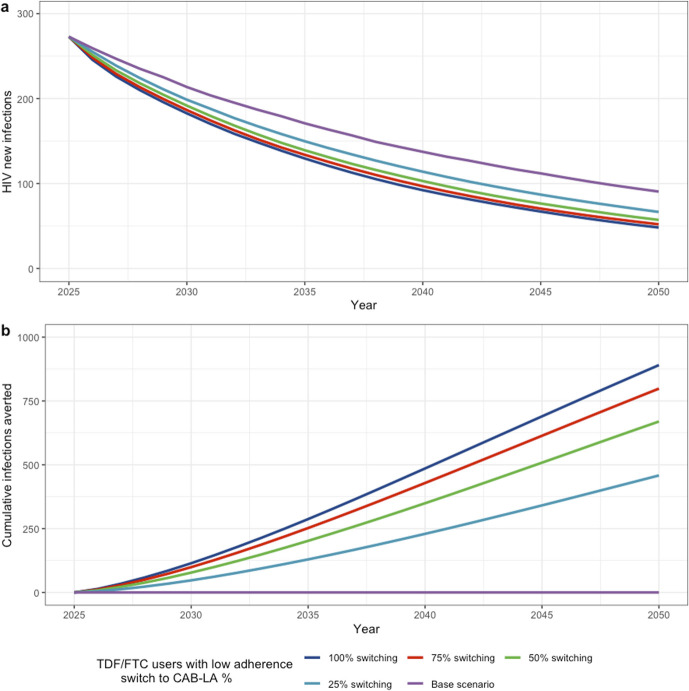
Projected population-level (A) MSM HIV new infections and (B) cumulative infections averted by expanding CAB-LA to current oral PrEP users with low adherence by different proportions of actual switchers for 25 years (2025–2050). Figure presents the percentage of MSM current oral PrEP users switching to CAB-LA. Lines show median across 178 fits.

Given an established sexual preference based on the PrEP use status so-called PrEP sorting,^28^ MSM who use PrEP would prefer to have sex with men who also use PrEP, and vice versa. This model included 2 main groups, and there was assortative mixing by sexual behaviors using PrEP-using status (non-PrEP users and PrEP users [both oral PrEP and CAB-LA]) as a behavioral proxy based on the annual number of condomless anal intercourse (CAI) with nonsteady partners. In this model, we assumed that PrEP users have a higher number of CAI with nonsteady partners than with non-PrEP users.^29^ The annual number of CAI with nonsteady partners was retrieved from the European MSM Internet Survey (EMIS-2017) with Dutch subsamples (N = 3851).^26^ More information on assortative mixing by sexual behaviors using oral PrEP status is shown in Figure 2, Supplemental Digital Content, http://links.lww.com/QAI/C679.

Assuming a daily-use regimen scenario, in this model, oral PrEP users were additionally stratified by their use adherence (estimated by the ratio of the number of days covered by PrEP dispensing during the total number of days),^1^ and was categorized as low (<50% of the days covered), medium (50%–74%), and high (≥75%). The real-world effectiveness of the TDF/FTC by use adherence was estimated as 18%, 69%, and 93%, respectively.^1^ Similarly, to account for real-world effectiveness rather than clinical efficacy, the baseline effectiveness of CAB-LA in our model was set at 91%, derived from the HPTN 083 trial data combining both efficacy and adherence data.^10,14^ However, the real-world effectiveness of CAB-LA in a nontrial setting is still unknown. Different factors may influence its real-world effectiveness, such as lower regimen adherence, higher regimen discontinuation, and poorer regimen administration.^8^ To account for these potential implementation challenges, we performed sensitivity analyses exploring a range of real-world CAB-LA effectiveness from 91% down to 61%. More key model parameters are given in Table 1.

We calibrated our model to the historical MSM HIV epidemic from 2017 to 2021 based on the size of the MSM population, the number of oral PrEP users, the number of MSM diagnosed with HIV, the estimated number of MSM living with HIV, the yearly number of new HIV diagnoses including the proportion diagnosed in a late stage (CD4<350 cells/µl) and advanced stage of infection (CD4<200 cells/µl), and the proportion of HIV diagnosed MSM receiving ART.^2^ Variables that were used for the model calibration are given in Table 1, Supplemental Digital Content, http://links.lww.com/QAI/C679. We accepted 178 of the 2 million simulations that best matched the Dutch HIV epidemic among MSM the most. All results are reported as the accepted simulations' median and interquartile range (IQR).

### Base-Case Scenario and CAB-LA Expansion Scenarios

The base-case scenario was simulated to project oral PrEP use and the HIV care continuum in the Netherlands in the absence of CAB-LA to represent the current and future HIV epidemic given the current TDF/FTC services and serve as counterfactual estimates when evaluating CAB-LA expansion scenarios.

We assume CAB-LA will be introduced in the Netherlands in 2025. We first explored the epidemiologic impact of CAB-LA by expanding CAB-LA to current nonoral PrEP users (both PrEP naïve and PrEP discontinued), while other current oral PrEP users were assumed to continue to use TDF/FTC. Given the limited capacity of PrEP provision in the Netherlands^30^ and a significantly lower intention to use long-acting PrEP regimens among the current nonoral PrEP users,^12^ we assumed the initiation rate of CAB-LA among current nonoral PrEP users is not higher than the assumed oral PrEP initiation rate among nonoral PrEP users. Among these scenarios, we first investigated whether the current nonoral PrEP users would adapt their sexual behavioral patterns (again) after initiating CAB-LA, namely if the annual number of CAI partners would be adapted as high as we assumed for PrEP users. Two scenarios were simulated and compared: current nonoral PrEP users who initiate CAB-LA would (1) adapt their sexual behavioral patterns similar to current oral PrEP users or (2) retain their sexual behavioral patterns similar to MSM who are not using PrEP. Also, given the established lower intention for CAB-LA among current nonoral PrEP users compared with current oral PrEP users in the Netherlands,^12^ we further explored the potential CAB-LA initiation rate among current nonoral PrEP users compared with the oral PrEP initiation rate (this ratio of initiation rates ranged from 0.25 to 1, where 1 indicates the same CAB-LA initiation rate as the oral PrEP initiation rate).

We then explored the epidemiologic impact of expanding CAB-LA to current oral PrEP users with different use adherence levels, assuming every MSM in the targeted subpopulation would switch to CAB-LA. Among scenarios of expanding CAB-LA to current oral PrEP users, it is not realistic to assume that every targeted MSM would switch to CAB-LA, given that there is still a proportion of current oral PrEP-using MSM in the Netherlands who did not show an intention and preference for LAI-PrEP.^12,31^ Therefore, we additionally explored the epidemiologic impact of CAB-LA when different proportions of the current oral PrEP users would actually switch to CAB-LA, ranging from 25% to 100%.

### Outcome Measures

The epidemiologic impact was measured as the cumulative new HIV infections averted for 25 years from the start of 2025, in comparison with the base-case scenario of no CAB-LA. We also assessed the new MSM HIV infections for 25 years to 2050 to investigate how expanding CAB-LA can further reduce the MSM HIV epidemic in the Netherlands.

## RESULTS

### Expanding CAB-LA to MSM Who Were Currently Not Using Oral PrEP

Overall, expanding CAB-LA to current nonoral PrEP users while the current oral PrEP users continue to use TDF/FTC can further reduce the HIV epidemic in the Netherlands. Yet, the projected epidemiologic impact remains suboptimal (Fig. 1).

The projected epidemiologic impact was strongly dependent on whether these PrEP naïve or MSM who discontinued oral PrEP would retain the sexual behavioral patterns. Our model forecasted that a cumulative 241 (5.5%) to 276 (6.3%) of the new HIV infections would be averted if these PrEP naïve or PrEP discontinued MSM would retain their previous sexual behavioral patterns and adapt to the average PrEP users' sexual behavioral patterns, respectively, for 25 years. The projected epidemiologic impact also depended on the ratio of CAB-LA initiation compared with the oral PrEP initiation rate, too. The projected cumulative averted HIV new infections percentage ranged from 1.8% to 5.5%, and from 2.1% to 6.3% in the scenarios of retained and adapted sexual behavioral patterns, respectively, for 25 years, when the ratio of rates of CAB-LA initiation over oral PrEP initiation increased from 0.25 to 1 (see Table 2, Supplemental Digital Content, http://links.lww.com/QAI/C679).

### Expanding CAB-LA to MSM Who Were Currently Using Oral PrEP

Switching to CAB-LA from oral PrEP will generally reduce the number of new infections. Importantly, however, providing CAB-LA to individuals who are currently very adherent to oral PrEP will result in a lower number of infections that can be averted, which is ascribed to the high effectiveness of frequent use of TDF/FTC (Fig. 1). Providing CAB-LA to oral PrEP users with low and medium adherence forecasted that 862 (19.7%) and 174 (4.0%) new HIV infections could be averted during 25 years, respectively.

We further modeled the epidemiologic impact of expanding CAB-LA to MSM who use oral PrEP with low adherence by different proportions of individuals actually switching to CAB-LA (Fig. 2). Overall, the projected epidemiologic impact of CAB-LA was strongly dependent on the actual switching proportion of oral PrEP users to CAB-LA. With a lower proportion of MSM who use oral PrEP with low adherence, the projected new HIV infections over time inclined and the cumulative infections averted declined. For 25 years, a total number of 51–67 new HIV infections were projected in 2050, with 862 (19.7%) down to 453 (10.1%) infections can be averted when the actual switching proportion decreases from 100% to 25% (Fig. 2).

**FIGURE 3.**
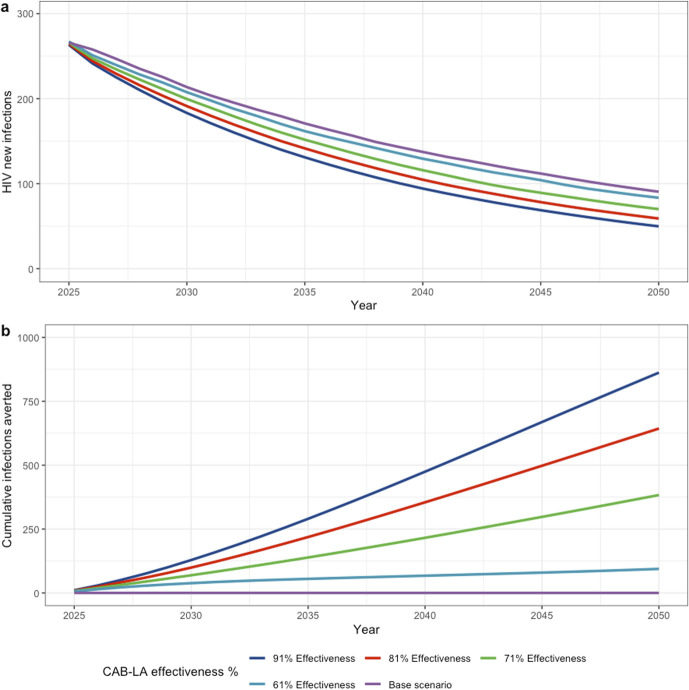
Projected population-level (A) MSM HIV new infections and (B) cumulative infections averted by expanding CAB-LA to current oral PrEP users with low adherence by different assumptions of real-world CAB-LA effectiveness for 25 years (2025–2050). Figure presents the percentage of MSM current oral PrEP users switching to CAB-LA. Lines show median across 178 fits.

Moreover, we modeled the epidemiologic impact of expanding CAB-LA to MSM who use oral PrEP with low adherence and lower real-world CAB-LA effectiveness (Fig. 3). Overall, the projected epidemiologic impact of CAB-LA was also strongly dependent on real-world CAB-LA effectiveness. In both scenarios of a lower switching proportion of targeted MSM with low adherence to oral PrEP would switch to CAB-LA and a lower real-world CAB-LA effectiveness, the projected new HIV infections over time inclined, and the cumulative infections averted declined. For 25 years, the total number of new HIV infections was projected to be up to 84 in 2050, with a decline in total number to 94 (2.1%) infections that can be averted during 25 years when the real-world CAB-LA effectiveness decreases to 61% (Fig. 3).

## DISCUSSION

Taking an innovative approach by identifying the most efficient CAB-LA targeting strategies, our model suggested that expanding CAB-LA among MSM in the Netherlands in 2025 can further reduce the current MSM HIV epidemic compared with using TDF/FTC only. Our model aligned with the hypothesis that using CAB-LA would be beneficial to MSM with low adherence to oral PrEP^12,18,32^ and suggested that prioritizing CAB-LA to MSM with low adherence to oral PrEP can further reduce the HIV epidemic. Even though other MSM subpopulations, including current nonoral PrEP users, would also benefit from using CAB-LA as the previous research hypothesized,^33^ the overall population impact was projected to be lower, and thus would not likely be an efficient approach in the context of the potential limited availability and affordability during the initial stage of CAB-LA introduction in the Dutch context.

Our findings propose a possible CAB-LA prioritizing strategy to prioritize CAB-LA to PrEP users who are currently partially adherent to oral regimens. Such a prioritization could avert 20% of all new HIV infections among MSM during the next 25 years. Our findings also suggest a strong epidemiologic impact when only 25%–75% of individuals with low adherence to oral PrEP switch to CAB-LA. Most importantly, despite a potential decrease in epidemiologic impact if real-world effectiveness is lower than clinical efficacy, our model still projected that CAB-LA would be highly beneficial for individuals with low oral PrEP adherence. This finding, therefore, reinforces our suggestion that CAB-LA should be prioritized for MSM with low adherence to oral PrEP. Yet, we would like to highlight that this proposed prioritizing strategy could be jeopardized by continued low adherence after a switch to CAB-LA. We acknowledge that for many MSM, particularly those struggling with daily oral PrEP, the transition to bimonthly injections may represent a lower overall barrier to adherence.^34,35^ However, empirical evidence from long-acting injectable ART indicates that “structural adherence” to clinic schedules is not guaranteed.^36^ Individuals may still miss or delay appointments because of travel, work, or other logistical challenges.^35^ Because the real-world effectiveness of CAB-LA as PrEP has not yet been established in large-scale implementation studies, it is crucial to consider how these missed visits might affect population-level benefits. Therefore, implementation strategies and interventions for these challenges should be included before the CAB-LA introduction, to ensure that adherence, discontinuation, and administration of CAB-LA are adequately emphasized and implemented, so they do not become barriers to effective prevention.

Our model also predicted the least epidemiologic impact when expanding CAB-LA to MSM who strictly adhere to the oral PrEP. However, we believe that the projection of slightly more HIV infections with the use of CAB-LA among MSM with high adherence to oral PrEP is likely because of numerical calculations based on our assumptions and may not reflect real-world practice. Considering the similarity in the assumed effectiveness between oral PrEP with high adherence (93%) and CAB-LA (91%), we suggest that MSM with high adherence to oral PrEP would derive similar benefits from using either oral PrEP or CAB-LA. Therefore, at the initial stage of CAB-LA introduction, it may not be recommended to prioritize CAB-LA to MSM who demonstrate high adherence to oral PrEP, because they could continue using oral PrEP. Of course, there are also other determinants relevant to such a decision, for example, increased individual risk exposure and the need for superior levels of protection, which may affect the decision to use CAB-LA otherwise.

Furthermore, based on our findings on current nonoral PrEP users, we do not recommend prioritizing CAB-LA to these current nonoral PrEP users at the initial phase of CAB-LA introduction in the Netherlands. One reason is the lower projected epidemiologic impact of expanding CAB-LA to current nonoral PrEP users compared with MSM with low oral PrEP adherence. In the beginning phase of the CAB-LA introduction, the CAB-LA is anticipated to be more costly and less available.^37^ Especially while this group of MSM showed a significantly lower intention and preference to use long-acting injectables in the Netherlands,^12^ with a lower and less efficient epidemiologic impact, CAB-LA should be prioritized over other groups that would benefit more from using CAB-LA with higher intention to use, so we could ensure higher CAB-LA adherence and retention, and maximize its public health benefits. Another reason is the limited PrEP provision capacity and PrEP care budget in the Netherlands through its National PrEP program.^38,39^ As a result, an extensive PrEP coverage expansion with CAB-LA is likely unrealistic in this country. Instead, a more tailored approach to maximize public health impact with similar overall PrEP coverage may be preferable and may be more likely to be implemented. Nevertheless, we still emphasize that noncurrent PrEP users should not be overlooked and neglected from benefiting from CAB-LA and should be targeted when CAB-LA becomes more available and affordable.

Our study has several strengths. First, we used the well-described Dutch data on the MSM HIV epidemic,^2^ and used the well-validated model, which described and predicted the MSM HIV epidemic with PrEP interventions.^21^ Second, in our model, we have also considered and included the coexistence of both oral PrEP and CAB-LA, because it is very unlikely that only 1 regimen would be available once CAB-LA becomes available. In addition, our model has applied the real-world effectiveness of oral PrEP based on users' adherence.^1^ Compared with other models, which assumed relatively high effectiveness^14–18^ for oral PrEP, our estimates should be devoid of underestimations of the advantage of CAB-LA over the oral regimens, and provide estimations of the epidemiologic impact of CAB-LA based on different oral PrEP use adherence levels.

There are several limitations to this analysis. First, our study assumed a daily regimen scenario of oral PrEP, and defined oral PrEP adherence based on use proportion.^1^ We acknowledge that defining adherence to PrEP regimens should be beyond the PrEP use proportion calculation,^1^ and should be understood within the context of the individual risk for an HIV infection and the use of other HIV prevention methods,^40^ such as using event-driven oral PrEP.^41^ Given that event-driven oral PrEP use has lower effectiveness than the daily use of oral PrEP under a high adherence condition,^1,41^ yet comes with similar suboptimal adherence issues,^42^ our estimations may, therefore, be considered conservative. Second, another limitation could be the use of the real-world effectiveness of oral PrEP by its use adherence levels estimated by Jourdain et al,^1^ which also requires the MSM oral PrEP users' adherence data to be collected at the same level. Owing to the lack of Dutch data on oral PrEP use adherence levels among MSM following the definitions, our study retrieved the French data on oral PrEP use adherence levels among MSM,^1^ and assumed it is comparable with the Dutch settings. However, given the similar meta-analytical evidence on the estimated suboptimal adherence to oral PrEP among MSM in the Global North,^7^ we believe the use of French data in the Dutch setting would not result in significant biases. Another limitation of our study is the reliance on behavioral data from the EMIS-2017 to parameterize sexual behavior and mixing. Although this represents the most comprehensive large-scale data set available for the MSM population in the Netherlands, we acknowledge that sexual behaviors and PrEP awareness may have shifted. Consequently, our estimations may be subject to bias if current mixing patterns differ significantly from 2017 levels. Nevertheless, EMIS-2017 remains the most robust source for the specific assortative mixing parameters required for our model calibration, and we believe it still provides a reliable proxy for behavioral patterns in this declining epidemic setting. We emphasize that future modeling studies should update these behavioral figures as newer, comprehensive data sets become available. Finally, our model overlooked the concerns of the potential drug resistance from using cabotegravir as a PrEP regimen,^43^ owing to a lack of relevant data. Future studies should, therefore, also include such information to refine and enhance the accuracy of the projections.

In conclusion, our findings suggest that the introduction of CAB-LA in addition to oral PrEP would further reduce the HIV epidemic and can be an important addition to reaching the 2030 goals for ending HIV as a global health threat. However, a prioritizing strategy should be applied to expand CAB-LA in a more tailored manner. Our proposed CAB-LA prioritizing strategy, focusing on prioritizing transitioning the current oral PrEP user with low adherence to CAB-LA, should apply to and should inform the HIV prevention programs in high-resource settings with similar declining HIV epidemics, such as other European contexts, to reach the full potential of CAB-LA and to further accelerate ending the HIV epidemic. However, more country-specific European evidence tailored to their specific epidemics is still needed to support further HIV prevention planning and strategies in Europe.

## Supplementary Material
