# Supplementary material for: Epidemiologic Impact of Prioritizing Long-Acting Injectable Cabotegravir to Men Who Have Sex With Men With Low Pre-Exposure Prophylaxis Use Adherence in the Netherlands: A Mathematical Modeling Study
Source: J Acquir Immune Defic Syndr. 2026 May 25;101(8):871–80. doi: 10.1097/QAI.0000000000003882 (PMC13372366; doi:10.1097/QAI.0000000000003882)
Supplement: Supplementary file 1 [file qai-101-871-s001.docx]

**Supplementary material**

**Epidemiological impact of prioritising long-acting injectable cabotegravir to MSM with low PrEP use adherence in the Netherlands: A mathematical modelling study**

**Authors:** Haoyi Wang^1,2§^, Kai J. Jonas^1^, David van de Vijver^2^

^§^Correspondence to: Haoyi Wang

Department of Work and Social Psychology, Maastricht University, Maastricht, 6200ER, the Netherlands

Phone: +31 43 38 84531

Email: [haoyi.wang@maastrichtuniversity.nl](mailto:haoyi.wang@maastrichtuniversity.nl)

# File S1 supplemental methods

## Mathematical transmission model

This study includes a compartmental deterministic mathematical transmission model that was developed for the HIV epidemic among men who have sex with men (MSM) in the Netherlands and Germany.^1,2^ We further developed this model based on the current oral PrEP using status and adherence among MSM to represent the current HIV epidemic and predict future HIV epidemic with/without long-acting injectable cabotegravir (CAB-LA) among MSM aged 15 years and older in the Netherlands. The schematic representation of the model is presented in figure S1.

**
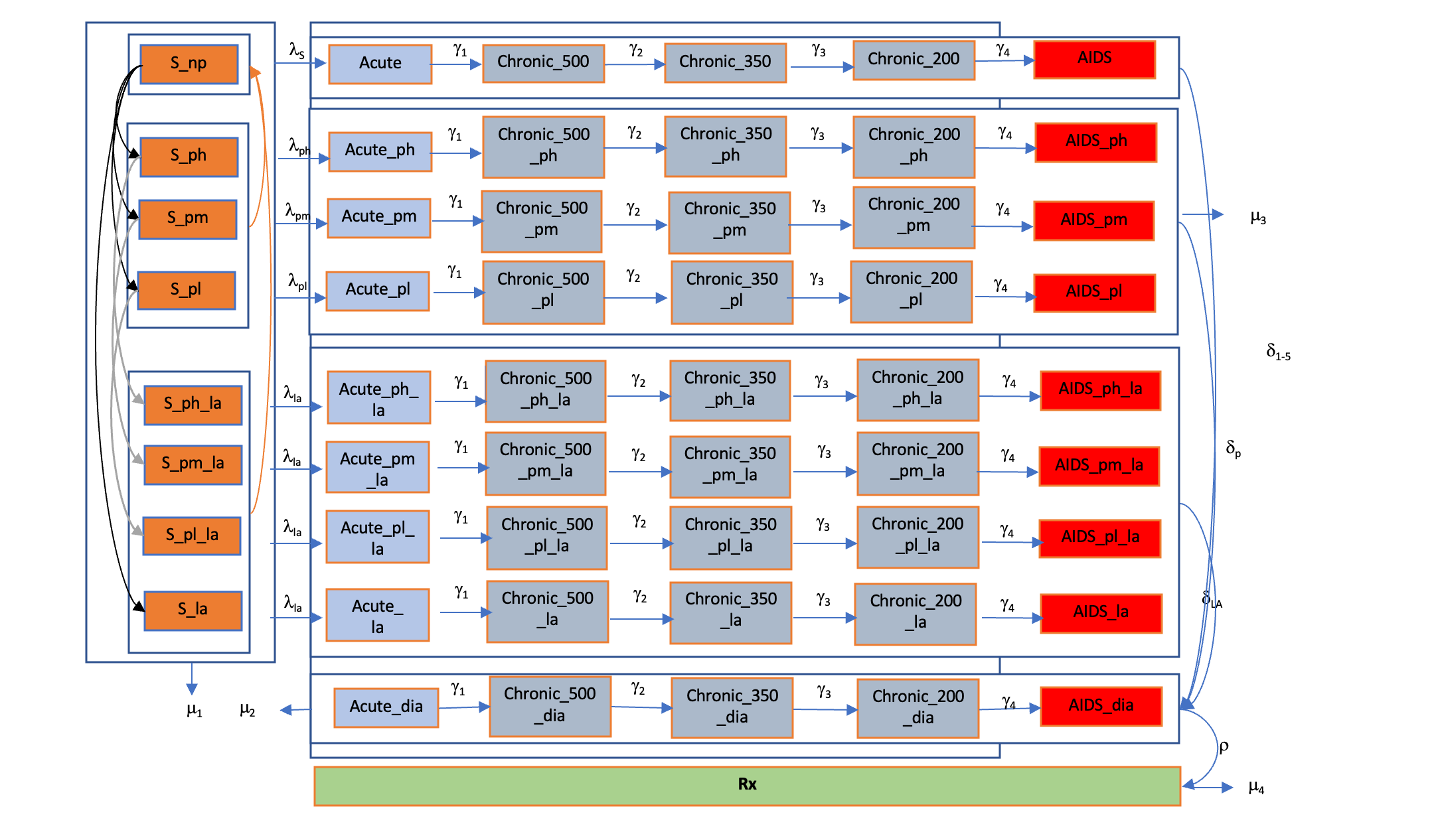
Figure S1 – Schematic representation of the compartmental deterministic. The state variables used in the equations are shown between brackets**.

This model consists of 54 compartments, which including:

1. Susceptible MSM for HIV infection who are not using oral PrEP including both PrEP naïve and PrEP discontinued (S_np)
2. Susceptible MSM for HIV infection who are using oral PrEP with high oral PrEP adherence (S_ph)
3. Susceptible MSM for HIV infection who are using oral PrEP with medium oral PrEP adherence (S_pm)
4. Susceptible MSM for HIV infection who are using oral PrEP with low oral PrEP adherence (S_pl)
5. Susceptible MSM for HIV infection who are using CAB-LA switching from using oral PrEP with high oral PrEP adherence (S_ph_la)
6. Susceptible MSM for HIV infection who are using CAB-LA switching from using oral PrEP with medium oral PrEP adherence (S_pm_la)
7. Susceptible MSM for HIV infection who are using CAB-LA switching from using oral PrEP with low oral PrEP adherence (S_pl_la)
8. Susceptible MSM for HIV infection who are using CAB-LA directly initiating from S_np (S_la)
9. S_np infected with HIV at the acute stage (Acute)
10. S_ph infected with HIV at the acute stage (Acute_ph)
11. S_pm infected with HIV at the acute stage (Acute_pm)
12. S_pl infected with HIV at the acute stage (Acute_pl)
13. S_ph_la infected with HIV at the acute stage (Acute_ph_la)
14. S_pm_la infected with HIV at the acute stage (Acute_pm_la)
15. S_pl_la infected with HIV at the acute stage (Acute_pl_la)
16. S_la infected with HIV at the acute stage (Acute_la)
17. Acute progressed to the first chronic stage CD4+ T-cell count>500 cells/ μL (Chronic_500)
18. Acute_ph progressed to the first chronic stage CD4+ T-cell count>500 cells/ μL (Chronic_500_ph)
19. Acute_pm progressed to the first chronic stage CD4+ T-cell count>500 cells/ μL (Chronic_500_pm)
20. Acute_pl progressed to the first chronic stage CD4+ T-cell count>500 cells/ μL (Chronic_500_pl)
21. Acute_ph_la progressed to the first chronic stage CD4+ T-cell count>500 cells/ μL (Chronic_500_ph_la)
22. Acute_pm_la progressed to the first chronic stage CD4+ T-cell count>500 cells/ μL (Chronic_500_pm_la)
23. Acute_pl_la progressed to the first chronic stage CD4+ T-cell count>500 cells/ μL (Chronic_500_pl_la)
24. Acute_la progressed to the first chronic stage CD4+ T-cell count>500 cells/ μL (Chronic_500 _la)
25. Chronic_500 progressed to the second chronic stage CD4+ T-cell count 350–500 cells/ μL (Chronic_350)
26. Chronic_500_ph progressed to the second chronic stage CD4+ T-cell count 350–500 cells/ μL (Chronic_350_ph)
27. Chronic_500_pm progressed to the second chronic stage CD4+ T-cell count 350–500 cells/ μL (Chronic_350_pm)
28. Chronic_500_pl progressed to the second chronic stage CD4+ T-cell count 350–500 cells/ μL (Chronic_350_pl)
29. Chronic_500_ph_la progressed to the second chronic stage CD4+ T-cell count 350–500 cells/ μL (Chronic_350_ph_la)
30. Chronic_500_pm_la progressed to the second chronic stage CD4+ T-cell count 350–500 cells/ μL (Chronic_350_pm_la)
31. Chronic_500_pl_la progressed to the second chronic stage CD4+ T-cell count 350–500 cells/ μL (Chronic_350_pl_la)
32. Chronic_500_la progressed to the second chronic stage CD4+ T-cell count 350–500 cells/ μL (Chronic_350_la)
33. Chronic_350 progressed to the third chronic stage CD4+ T-cell count 200-350 cells/ μL (Chronic_200)
34. Chronic_350_ph progressed to the third chronic stage CD4+ T-cell count 200-350 cells/ μL (Chronic_200_ph)
35. Chronic_350_pm progressed to the third chronic stage CD4+ T-cell count 200-350 cells/ μL (Chronic_200_pm)
36. Chronic_350_pl progressed to the third chronic stage CD4+ T-cell count 200-350 cells/ μL (Chronic_200_pl)
37. Chronic_350_ph_la progressed to the third chronic stage CD4+ T-cell count 200-350 cells/ μL (Chronic_200_ph_la)
38. Chronic_350_pm_la progressed to the third chronic stage CD4+ T-cell count 200-350 cells/ μL (Chronic_200_pm_la)
39. Chronic_350_pl_la progressed to the third chronic stage CD4+ T-cell count 200-350 cells/ μL (Chronic_200_pl_la)
40. Chronic_350_la progressed to the third chronic stage CD4+ T-cell count 200-350 cells/ μL (Chronic_200_la)
41. Chronic_200 progressed to the AIDS stage (AIDS)
42. Chronic_200_ph progressed to the AIDS stage (AIDS_ph)
43. Chronic_200_pm progressed to the AIDS stage (AIDS_pm)
44. Chronic_200_pl progressed to the AIDS stage (AIDS_pl)
45. Chronic_200_ph_la progressed to the AIDS stage (AIDS_ph_la)
46. Chronic_200_pm_la progressed to the AIDS stage (AIDS_pm_la)
47. Chronic_200_pl_la progressed to the AIDS stage (AIDS_pl_la)
48. Chronic_200_la progressed to the AIDS stage (AIDS_la)
49. MSM living with HIV diagnosed at Acute stage (Acute_dia)
50. MSM living with HIV diagnosed at the first chronic stage CD4+ T-cell count>500 cells/ μL (Chronic_500_dia)
51. MSM living with HIV diagnosed at the second chronic stage CD4+ T-cell count 350–500 cells/ μL (Chronic_350_dia)
52. MSM living with HIV diagnosed at the third chronic stage CD4+ T-cell count 200-350cells/ μL (Chronic_200_dia)
53. MSM living with HIV diagnosed at the AIDS stage (AIDS_dia)
54. MSM diagnosed with HIV and linked to treatment (Rx)

The parameters used in the equations are added next to the arrows that indicate the rate of change between the different compartments of the model. We assume that MSM can only start using PrEP/CAB-LA when they are not infected with HIV. MSM can become infected with HIV despite the use of PrEP/CAB-LA.

## Equations of the transmission model

The model consists of 49 ordinary differential equations, including equations that describe MSM that are not infected with HIV that use oral PrEP, CAB-LA or do not use PrEP, equations that describe disease progression, equations that describe the force of infection (or the rate by which MSM become infected) [3] and equations that describe sexual behavioural mixing matrix using PrEP using status as behavioural proxy based on the number of condomless anal intercourse (CAI) with non-steady partners. The equations are summarized below.

### Ordinary differential equations for MSM not infected with HIV

MSM not infected with HIV are divided into MSM that are not on PrEP, denoted as S_np_; MSM using oral PrEP, which were sub-divided by their oral PrEP adherence into MSM using oral PrEP with high adherence (S_ph_), medium adherence (S_pm_) and low adherence (Spl), given the behavioural stability, we assumed a static adherence status; and MSM using CAB-LA by their route of initiation into MSM using CAB-LA initiated from oral PrEP users with high adherence (S_phla_), oral PrEP users with medium adherence (S_pmla_), oral PrEP users with low adherence (S_plla_), and direct initiate from non-PrEP users (both PrEP naïve and discontinued, S_la_). Susceptible MSM have a mortality rate μ_1._^3,4^ MSM that become sexually active enter the group of susceptible MSM not using PrEP at a rate Α. Values for the parameter Α have been calibrated to the Dutch MSM population (Table S1).

Susceptible MSM can start PrEP at a rate Θ_p_. Θ_p_ is the sum rate of the rates Θ_ph_, Θ_pm_, Θ_pl_ for MSM using oral PrEP with high, medium and low adherence respectively, which were adjusted by the proportions of MSM using oral PrEP with different adherence in real life ^5^. parameter Α have been calibrated to the Dutch MSM population (Table S1). Susceptible MSM can discontinue PrEP at a rate Φ_p_ and can also start CAB-LA at a rate Θ_la_ and discontinue CAB-LA at a rate Φ_la_. Given no clinical/real-world data on CAB-LA was available in the Netherlands, Φ_la_ was retrieved from HPTN083 data.^6^ Θ_la_ was adjusted by the CAB-LA/oral PrEP initiation ratio ($\frac{\Theta_{\mathrm{la}}}{\Theta_{p}}$) of 1, 0.75, 0.5 and 0.25, given PrEP naïve and discontinued MSM are less likely to initial CAB-LA compared to current oral PrEP users in the Netherlands.^7^ Oral PrEP users can also start CAB-LA directly at Θ_phla_, Θ_pmla_, Θ_plla_ which indicates a proportion of 100%, 75%, 50% and 25% for MSM using oral PrEP with high, medium and low adherence, respectively.

The force of infection, denoted as λ_S_ for MSM not using PrEP; λ_ph_, λ_pm_, and λ_pl_ for, MSM using oral PrEP with high, medium and low adherence, respectively; and λ_phla_, λ_pmla_, λ_plla_, and λ_la_ for MSM using CAB-LA initiated from MSM using oral PrEP with high, medium and low adherence and directly from MSM not using PrEP, respectively, represents the rate by which MSM become infected with HIV.

S_np_’ = Α - S_np_*(Θ_ph_ + Θ_pm_ + Θ_pl_ + Θ_la_ + λ_S_ + μ_1_) + Φ_p_* (S_ph_ + S_pm_ + S_pl_) + Φ_la_* (S_phla_ + S_pmla_ + S_plla_+ S_la_) (1)

S_ph_’ = S_np_*Θ_ph_ – S_ph_*( Φ_p_ + Θ_phla_ + λ_ph_ + μ_1_) (2)

S_pm_’ = S_np_*Θ_pm_ – S_pm_*( Φ_p_ + Θ_pmla_ + λ_pm_ + μ_1_) (3)

S_pl_’ = S_np_*Θ_pl_ – S_pl_*( Φ_p_ + Θ_plla_ + λ_ph_ + μ_1_) (4)

S_phla_’ = S_ph_*Θ_phla_ – S_phla_*( Φ_la_ + λ_phla_ + μ_1_) (5)

S_pmla_’ = S_pm_*Θ_pmla_ – S_pmla_*( Φ_la_ + λ_pmla_ + μ_1_) (6)

S_plla_’ = S_pl_*Θ_plla_ – S_plla_*( Φ_la_ + λ_plla_ + μ_1_) (7)

S_la_’ = S_np_*Θ_la_ – S_la_*( Φ_la_ + λ_la_ + μ_1_) (8)

### Equations for undiagnosed HIV infected MSM not using PrEP

MSM that become infected with HIV but that have not been diagnosed yet (denoted with *H,* including compartment 9, 17, 25, 33 and 41, in equations 9 through 10), progress through five stages of infection that are denoted by the parameter σ. These stages of infection are the acute stage (σ = 1), the chronic stages (σ = 2 if the CD4 cell count is > 500 cells/µl and σ = 4 for a CD4 between 200 and 350 cells/µl) and the AIDS stage (σ = 5). MSM progress through infection at a rate γ_σ_ that depends on the stage of infection σ.^8^ HIV diagnositics were not available before 1985. MSM are diagnosed at a rate δ_σ_ that has been calibrated to the proportion of MSM that are diagnosed at a particular CD4 threshold as reported by the Stichting HIV Monitoring in the Netherlands.^9^ The mortality rate depends on the stage of infection, we assumed the mortality rates are comparable between acute stage and any chronic stage (denoted as μ_2_), and are different from AIDS stage (denoted as μ_3_).^3,4^

H_σ_’ = λ_S_* S_np_ - H_σ_*(δ_σ_+ γ_σ_+ µ_2_) … for σ = 1 (9)

H_σ_’ = H_σ-1_*γ_σ-1_ - H_σ_*(δ_σ_+ γ_σ_+ µ_2_) … for σ = 2,3,4 (10)

H_σ_’ = H_σ-1_*γ_σ-1_ - H_σ_*(δ_σ_ + µ_3_) … for σ = 5 (11)

### Equations for undiagnosed HIV infected MSM using oral PrEP and CAB-LA

The equations used to model MSM that become infected with HIV despite the use of oral PrEP with different adherences and CAB-LA (denoted as *H_ph_, H_pm_, H_pl_, H_phla_, H_pmla_, H_plla_*, and *H_la_*, respectively, (including compartment 18-24, 26-32, 34-40, 42-48), in equations 12 through 32), are comparable to the equations used to model HIV-infected MSM that do not use PrEP (equations 9 through 11). MSM using oral PrEP are assumed to be tested for HIV at a rate (denoted as δ_p_). MSM using CAB-LA are assumed to be tested for HIV every two months before their injection at a rate (denoted as δ_la_).

For MSM using oral PrEP with high adherence:

H_phσ_’ = λ_ph_* S_ph_ - H_phσ_*( + γ_σ_+ µ_2_) … for σ = 1 (12)

H_phσ_’ = H_phσ-1_*γ_σ-1_ - H_phσ_*(δ_p_+ γ_σ_+ µ_2_) … for σ = 2,3,4 (13)

H_phσ_’ = H_phσ-1_*γ_σ-1_ - H_phσ_*(δ_p_ + µ_3_) … for σ = 5 (14)

For MSM using oral PrEP with medium adherence:

H_pmσ_’ = λ_pm_* S_pm_ - H_pmσ_*(δ_p_+ γ_σ_+ µ_2_) … for σ = 1 (15)

H_pmσ_’ = H_phσ-1_*γ_σ-1_ - H_pmσ_*(δ_p_+ γ_σ_+ µ_2_) … for σ = 2,3,4 (16)

H_pmσ_’ = H_pmσ-1_*γ_σ-1_ - H_pmσ_*(δ_p_ + µ_3_) … for σ = 5 (17)

For MSM using oral PrEP with low adherence:

H_plσ_’ = λ_pl_* S_pl_ - H_plσ_*(δ_p_+ γ_σ_+ µ_2_) … for σ = 1 (18)

H_plσ_’ = H_phσ-1_*γ_σ-1_ - H_plσ_*(δ_p_+ γ_σ_+ µ_2_) … for σ = 2,3,4 (19)

H_plσ_’ = H_plσ-1_*γ_σ-1_ - H_plσ_*(δ_p_ + µ_3_) … for σ = 5 (20)

For MSM using CAB-LA switching from using oral PrEP with high oral PrEP adherence:

H_phlaσ_’ = λ_phla_* S_phla_ - H_phlaσ_*(δ_la_+ γ_σ_+ µ_2_) … for σ = 1 (21)

H_phlaσ_’ = H_phlaσ-1_*γ_σ-1_ - H_phlaσ_*(δ_la_+ γ_σ_+ µ_2_) … for σ = 2,3,4 (22)

H_phlaσ_’ = H_phlaσ-1_*γ_σ-1_ - H_phlaσ_*(δ_la_ + µ_3_) … for σ = 5 (23)

For MSM using CAB-LA switching from using oral PrEP with medium oral PrEP adherence:

H_pmlaσ_’ = λ_pmla_* S_pmla_ - H_pmlaσ_*(δ_la_+ γ_σ_+ µ_2_) … for σ = 1 (24)

H_pmlaσ_’ = H_pmlaσ-1_*γ_σ-1_ - H_pmlaσ_*(δ_la_+ γ_σ_+ µ_2_) … for σ = 2,3,4 (25)

H_pmlaσ_’ = H_pmlaσ-1_*γ_σ-1_ - H_pmlaσ_*(δ_la_ + µ_3_) … for σ = 5 (26)

For MSM using CAB-LA switching from using oral PrEP with low oral PrEP adherence:

H_pllaσ_’ = λ_plla_* S_plla_ - H_pllaσ_*(δ_la_+ γ_σ_+ µ_2_) … for σ = 1 (27)

H_pllaσ_’ = H_pllaσ-1_*γ_σ-1_ - H_pllaσ_*(δ_la_+ γ_σ_+ µ_2_) … for σ = 2,3,4 (28)

H_pllaσ_’ = H_pllaσ-1_*γ_σ-1_ - H_pllaσ_*(δ_la_ + µ_3_) … for σ = 5 (29)

For MSM using CAB-LA directly initiating from MSM not using oral PrEP:

H_laσ_’ = λ_la_* S_la_ - H_laσ_*(δ_la_+ γ_σ_+ µ_2_) … for σ = 1 (30)

H_laσ_’ = H_laσ-1_*γ_σ-1_ - H_laσ_*(δ_la_+ γ_σ_+ µ_2_) … for σ = 2,3,4 (31)

H_laσ_’ = H_laσ-1_*γ_σ-1_ - H_laσ_*(δ_la_ + µ_3_) … for σ = 5 (32)

### Equations for HIV untreated infected MSM that have been diagnosed

MSM that are diagnosed with HIV but that are not treated with antiretroviral drugs (yet) are denoted as *D* (including compartment 49-53, equations 33 through 35). Diagnosed MSM start treatment at a rate ρ_σ_. Antiretroviral drugs were not available before 1996 in the Netherlands. The rate ρ_σ_ depends on the stage of infection σ to reflect past changes in the CD4 cell count at which treatment was initiated. Treatments were only available to MSM diagnosed with HIV at AIDS stage from 1996 to 2006. Treatments became available to MSM diagnosed with HIV at the third chronic stage CD4+ T-cell count 200-350 cells from 2007, and available to all MSM diagnosed with from 2012 regardless of the stage of infection.

D_σ_’ = δ_σ_* H_σ_ + δ_p_*( H_ph_+H_pm_+H_pl_) + δ_la_*( H_phla_+H_pmla_+H_plla_+H_la_) - D_σ_*( ρ_σ_+ γ_σ_+ µ_2_)… for σ = 1 (33)

D_σ_’ = δ_σ_* H_σ_ + δ_p_*( H_ph_+H_pm_+H_pl_) + γ_σ-1_* D_σ-1_ - D_σ_*( ρ_σ_+ γ_σ_+ µ_2_)… for σ = 2,3,4 (34)

D_σ_’ = δ_σ_* H_σ_ + δ_p_*( H_ph_+H_pm_+H_pl_) + γ_σ-1_* D_σ-1_ - D_σ_*( ρ_σ_+ µ_3_)… for σ = 5 (35)

### Equations for MSM using antiretroviral drug treatment

MSM that use antiretroviral drugs are represented by the state variable *Rx* (including compartment 54, equation 36). People treated with antiretroviral drug treatment are assumed to have the same mortality as the general population ^10^.

Rx’ = $\sum_{\sigma=1}^{5} \rho_{\sigma}*D_{\sigma} - Rx* \mu_{1}$ (36)

### Equations for the force of infection

The force of infection depends on the contact rate of CAI partners ($c_{i}$) for MSM’s PrEP using status $i$, where $i$=np denotes MSM not using PrEP, and $i$=p denotes MSM using PrEP (both oral PrEP and CAB-LA); and the probability by which these MSM form a CAI sexual relationship with an MSM with sexual activity $i$ as determined by probability of that ${MSM}_{i}$ being living with HIV. The rate of infection also depends on the infectivity k of the different stages of infection σ. The model uses four different parameters of effectiveness of PrEP regimens to adjust the forces of infections for MSM using PrEP, including E_ph_, E_pm_, and E_pl_, for MSM using oral PrEP with low high, medium and low adherence respectively, and E_la_ for MSM using CAB-LA. In the Netherlands, MSM who are diagnosed with HIV will be linked to treatment immediately. Given the strong evidence of Undetectable equals Untransmutable (U=U),^11^ and viral suppression in the Netherlands is very high (96%),^9^ we assumed that MSM with HIV who were linked to treatment was not infectious.

We also applied an assortative mixing matrix by sexual behaviours using PrEP-using status (both oral PrEP and CAB-LA) as a behavioural proxy based on the annual number of condomless anal intercourse (CAI) partners, see Figure S2. Given the evidence that the number of CAI partners increases significantly after PrEP initiation,^12^ but remains similar regardless of users’ adherence,^13^ we assumed that MSM’s sexual behaviours are only dependent on their oral PrEP use status but remain independent from their adherence to oral PrEP. Given an established sexual preference based on the PrEP use status so-called PrEP sorting,^14,15^ MSM who use oral PrEP would prefer to have sex with men who also use oral PrEP, and vice versa, we implemented a weak probability statement (formula 37) in the assortative mixing matrix, assuming the probability of MSM who are currently using oral PrEP are more likely to have sex with MSM who are also currently using oral PrEP, and MSM who are not using PrEP are more likely to have sex with MSM who are not using PrEP.

$P\left( c_{i}*p_{ii}>0.5 \right)=\frac{2}{3},i=MSM belong to the same sexual behavioural group i$ (37)

Where $c_{i}$ is the average CAI non-steady partners of ${MSM}_{i}$, $p_{ii}$ is the probability of ${MSM}_{i}$having CAI with ${MSM}_{i}$ from the same group, $p_{ij}$ is the probability of ${MSM}_{i}$having CAI with ${MSM}_{j}$ from the different group.

C_n1_ = C_np_*$p_{ii}$ (38)

C_n2_ = C_np_*$p_{ij}$ (39)

C_p1_ = C_p_*$p_{ij}$ (40)

C_p2_ = C_p_*$p_{ii}$ (41)

**Figure S2 – Sexual behaviours mixing matrix using PrEP-using status (both oral PrEP and CAB-LA) as a behavioural proxy**


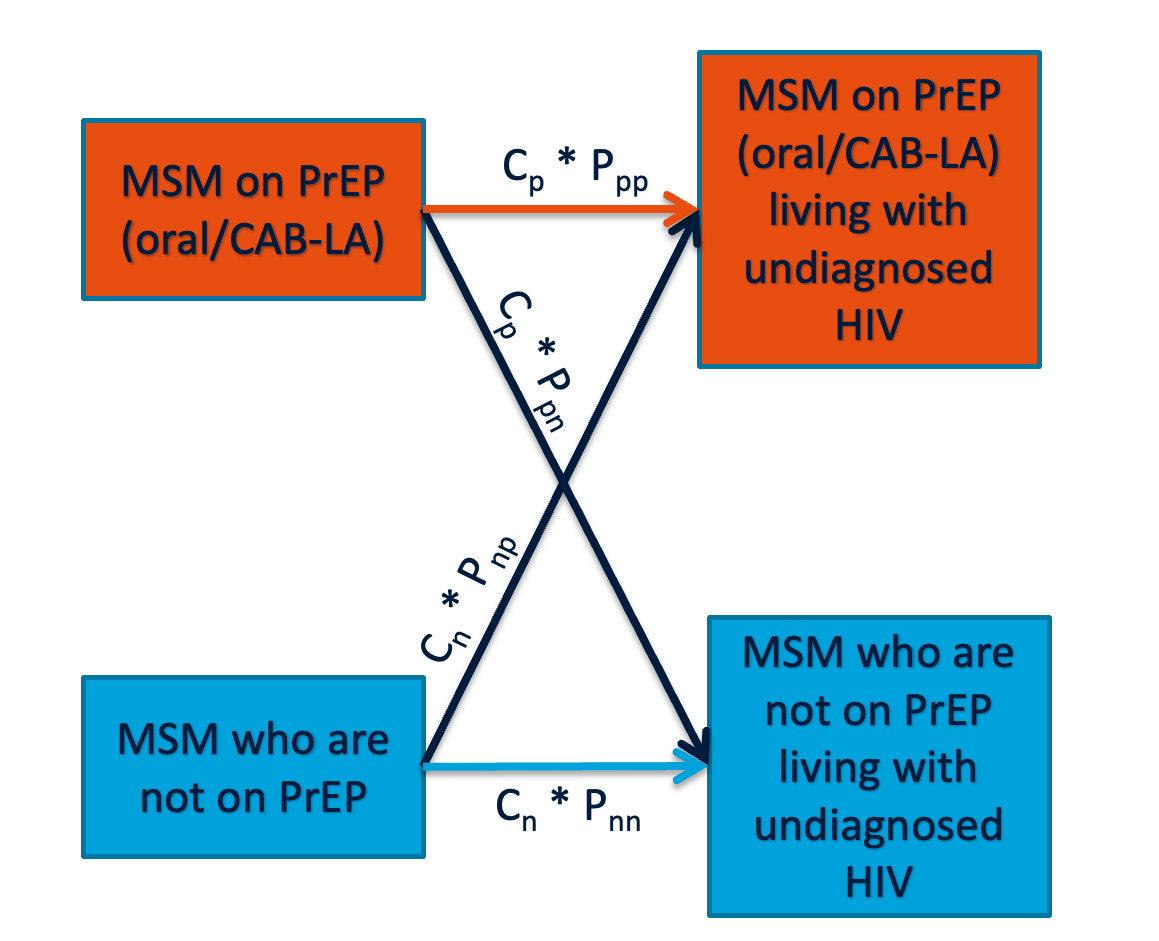


^Note: Ci: average contact number of MSMi (# CAI partners). Pij: probability of MSMi having CAI with each group of MSMj^

λ_s_ = C_n1_*$\sum_{\sigma=1}^{5} k_{\sigma}*\frac{k_{\sigma}*H_{\sigma}}{H_{N}}$ + C_n2_*[$\sum_{\sigma=1}^{5} (\frac{k_{\sigma}*(H_{ph\sigma+}H_{pm\sigma}+H_{pl\sigma}+H_{phla\sigma}{+H}_{pmla\sigma}{+H}_{plla\sigma}{+H}_{la\sigma})}{H_{\mathrm{pN}}+H_{\mathrm{laN}}}$)] (42)

λ_ph_ = C_p1_*$\sum_{\sigma=1}^{5} k_{\sigma}*\frac{k_{\sigma}*H_{\sigma}}{H_{N}}$ + C_p2_*[$\sum_{\sigma=1}^{5} (\frac{k_{\sigma}*(H_{ph\sigma+}H_{pm\sigma}+H_{pl\sigma}+H_{phla\sigma}{+H}_{pmla\sigma}{+H}_{plla\sigma}{+H}_{la\sigma})}{H_{\mathrm{pN}}+H_{\mathrm{laN}}}$)]* (1-E_ph_) (43)

λ_pm_ = C_p1_*$\sum_{\sigma=1}^{5} k_{\sigma}*\frac{k_{\sigma}*H_{\sigma}}{H_{N}}$ + C_p2_*[$\sum_{\sigma=1}^{5} (\frac{k_{\sigma}*(H_{ph\sigma+}H_{pm\sigma}+H_{pl\sigma}+H_{phla\sigma}{+H}_{pmla\sigma}{+H}_{plla\sigma}{+H}_{la\sigma})}{H_{\mathrm{pN}}+H_{\mathrm{laN}}}$)]* (1-E_pm_) (44)

λ_pl_ = C_p1_*$\sum_{\sigma=1}^{5} k_{\sigma}*\frac{k_{\sigma}*H_{\sigma}}{H_{N}}$ + C_p2_*[$\sum_{\sigma=1}^{5} (\frac{k_{\sigma}*(H_{ph\sigma+}H_{pm\sigma}+H_{pl\sigma}+H_{phla\sigma}{+H}_{pmla\sigma}{+H}_{plla\sigma}{+H}_{la\sigma})}{H_{\mathrm{pN}}+H_{\mathrm{laN}}}$)]* (1-E_pl_) (45)

λ_phla_ = C_p1_*$\sum_{\sigma=1}^{5} k_{\sigma}*\frac{k_{\sigma}*H_{\sigma}}{H_{N}}$ + C_p2_*[$\sum_{\sigma=1}^{5} (\frac{k_{\sigma}*(H_{ph\sigma+}H_{pm\sigma}+H_{pl\sigma}+H_{phla\sigma}{+H}_{pmla\sigma}{+H}_{plla\sigma}{+H}_{la\sigma})}{H_{\mathrm{pN}}+H_{\mathrm{laN}}}$)]* (1-E_la_) (46)

λ_pmla_ = C_p1_*$\sum_{\sigma=1}^{5} k_{\sigma}*\frac{k_{\sigma}*H_{\sigma}}{H_{N}}$ + C_p2_*[$\sum_{\sigma=1}^{5} (\frac{k_{\sigma}*(H_{ph\sigma+}H_{pm\sigma}+H_{pl\sigma}+H_{phla\sigma}{+H}_{pmla\sigma}{+H}_{plla\sigma}{+H}_{la\sigma})}{H_{\mathrm{pN}}+H_{\mathrm{laN}}}$)]* (1-E_la_) (47)

λ_plla_ = C_p1_*$\sum_{\sigma=1}^{5} k_{\sigma}*\frac{k_{\sigma}*H_{\sigma}}{H_{N}}$ + C_p2_*[$\sum_{\sigma=1}^{5} (\frac{k_{\sigma}*(H_{ph\sigma+}H_{pm\sigma}+H_{pl\sigma}+H_{phla\sigma}{+H}_{pmla\sigma}{+H}_{plla\sigma}{+H}_{la\sigma})}{H_{\mathrm{pN}}+H_{\mathrm{laN}}}$)]* (1-E_la_) (48)

λ_la_ = C_p1_*$\sum_{\sigma=1}^{5} k_{\sigma}*\frac{k_{\sigma}*H_{\sigma}}{H_{N}}$ + C_p2_*[$\sum_{\sigma=1}^{5} (\frac{k_{\sigma}*(H_{ph\sigma+}H_{pm\sigma}+H_{pl\sigma}+H_{phla\sigma}{+H}_{pmla\sigma}{+H}_{plla\sigma}{+H}_{la\sigma})}{H_{\mathrm{pN}}+H_{\mathrm{laN}}}$)]* (1-E_la_) (49)

Where $H_{N}$ is the sum of MSM not using PrEP regardless of HIV status, $H_{\mathrm{pN}}$ is the sum of MSM using oral PrEP regardless of HIV status and $H_{\mathrm{laN}}$ is the sum of MSM not using CAB-LA regardless of HIV status.

## Model calibration

We calibrated our model to the historical epidemic based on: the estimated size of MSM population, the number of oral PrEP users, the number of MSM diagnosed with HIV, the estimated number of MSM living with HIV, the yearly number of new HIV diagnoses including the proportion diagnosed in a late stage (CD4<350 cells/mm^3) and advanced stage of infection (CD4<200 cells/mm^3), and the proportion of HIV diagnosed MSM receiving ART, variables that were used for model calibration can be found in Table S1. Latin Hypercube sampling was used to select two million different combinations of parameters from these ranges, running the model from 1981 to 2021 using each parameter combination in turn, comparing model outputs (demographic, epidemiological, HIV care continuum, PrEP use) to calibration data, and selecting those combinations of parameters (model ‘fits’) for which model outputs fell within all the pre-determined ranges that most match with the Dutch HIV epidemic among MSM (Table S1).

**Table S1 Variables used to calibrate and accept simulation using Latin Hypercube sampling techniques.**

| Parameter used for calibration | | Data in real world | Values accepted in calibration range | Source |
| --- | --- | --- | --- | --- |
| MSM population 2018-2020 | | 131515 | 130000 - 200000 | ^16^ |
| The number of oral PrEP users 2022 | | 8500 | 7000 - 11000 | ^7,17^ |
| Number of MSM diagnosed with HIV 2020 | | 13876 | 12500 - 15000 | ^9^ |
| The estimated number of MSM living with HIV 2020 | | 14500 | 12000 - 16000 | ^9^ |
| Number of new diagnosis amiong MSM | |  |  | ^9^ |
|  | 2018 | 446 | 300 - 600 |  |
|  | 2019 | 340 | 200 - 500 |  |
|  | 2020 | 257 | 100 - 400 |  |
| Proportion late diagnosis | | 42% | 37% - 47% | ^9^ |
| Proportion advanced diagnosis | | 22% | 17% - 27% | ^9^ |
| The proportion of HIV diagnosed MSM receiving ART | |  |  | ^9^ |
|  | 2020 | 92% | 87% - 98% |  |
|  | 2021 | 96% | 91% - 100% |  |

^Note: A total of 178 simulation were accepted (out of 2 million simulations run).^

**S2 Table. Population-level projected cumulative averted new HIV infection proportion by expanding CAB-LA to current non-oral PrEP users by different sexual behaviours assumptions and ratio of rates of CAB-LA/ oral PrEP initiation.**

| Sexual behaviours assumption | Expand CAB-LA to current non-oral PrEP users(ratio of rates of CAB-LA/Oral PrEP initiation) | Time period | **Median averted HIV infections % (IQR) among MSM populations compared to base-case scenario** |  |
| --- | --- | --- | --- | --- |
|  |  |  |  |  |
|  |  |  |  |  |
| CAB-LA users remain sexual behaviours as non-PrEP users | 1 | Over 25 years | 5.5 |  |
|  |  | (2025-2050) | (3.7 - 7.5) |  |
|  | 0.75 | Over 25 years | 4.5 |  |
|  |  | (2025-2050) | (3.0 - 6.2) |  |
|  | 0.50 | Over 25 years | 3.3 |  |
|  |  | (2025-2050) | (2.1 - 4.5) |  |
|  | 0.25 | Over 25 years | 1.8 |  |
|  |  | (2025-2050) | (1.1 - 2.5) |  |
| CAB-LA users have the same sexual behaviours as PrEP users | 1 | Over 25 years | 6.3 |  |
|  |  | (2025-2050) | (4.3 - 8.4) |  |
|  | 0.75 | Over 25 years | 5.1 |  |
|  |  | (2025-2050) | (3.3 - 6.9) |  |
|  | 0.50 | Over 25 years | 3.7 |  |
|  |  | (2025-2050) | (2.4 - 5.0) |  |
|  | 0.25 | Over 25 years | 2.1 |  |
|  |  | (2025-2050) | (1.3 - 2.9) |  |

^Note: CAB-LA = long-acting injectable cabotegravir. IQR = interquartile range. MSM = men who have sex with men. PrEP = Pre-exposure prophylaxis.^

# Reference

1. Nichols BE, Boucher CAB, van der Valk M, Rijnders BJA, van de Vijver DAMC. Cost-effectiveness analysis of pre-exposure prophylaxis for HIV-1 prevention in the Netherlands: a mathematical modelling study. *Lancet Infect Dis* 2016; **16**(12): 1423-9.

2. van de Vijver D, Richter AK, Boucher CAB, et al. Cost-effectiveness and budget effect of pre-exposure prophylaxis for HIV-1 prevention in Germany from 2018 to 2058. *Euro Surveill* 2019; **24**(7).

3. Nakagawa F, Lodwick RK, Smith CJ, et al. Projected life expectancy of people with HIV according to timing of diagnosis. *Aids* 2012; **26**(3): 335-43.

4. WHO. Life expectancy and Healthy life expectancy

Data by country. 2022. <https://apps.who.int/gho/data/node.main.688?lang=en>.

5. Jourdain H, de Gage SB, Desplas D, Dray-Spira R. Real-world effectiveness of pre-exposure prophylaxis in men at high risk of HIV infection in France: a nested case-control study. *Lancet Public Health* 2022; **7**(6): e529-e36.

6. Landovitz RJ, Donnell D, Clement ME, et al. Cabotegravir for HIV Prevention in Cisgender Men and Transgender Women. *N Engl J Med* 2021; **385**(7): 595-608.

7. Wang H, Zimmermann HML, van de Vijver D, Jonas KJ. Intention and preference for long-acting injectable PrEP among MSM in the Netherlands: a diffusion of innovation approach. *medRxiv* 2022: 2022.11.11.22282218.

8. Lodi S, Phillips A, Touloumi G, et al. Time from human immunodeficiency virus seroconversion to reaching CD4+ cell count thresholds <200, <350, and <500 Cells/mm³: assessment of need following changes in treatment guidelines. *Clin Infect Dis* 2011; **53**(8): 817-25.

9. van Sighem AI, Wit F, Boyd A, Smit C, Matser A, Reiss P. Monitoring Report 2022. Human Immunodeficiency Virus (HIV) Infection in the Netherlands. Amsterdam: Stichting HIV Monitoring, 2022. 2022. <https://www.hiv-monitoring.nl/en/resources/monitoring-reports>.

10. Life expectancy of individuals on combination antiretroviral therapy in high-income countries: a collaborative analysis of 14 cohort studies. *Lancet* 2008; **372**(9635): 293-9.

11. Eisinger RW, Dieffenbach CW, Fauci AS. HIV Viral Load and Transmissibility of HIV Infection: Undetectable Equals Untransmittable. *Jama* 2019; **321**(5): 451-2.

12. Oldenburg CE, Nunn AS, Montgomery M, et al. Behavioral Changes Following Uptake of HIV Pre-exposure Prophylaxis Among Men Who Have Sex with Men in a Clinical Setting. *AIDS Behav* 2018; **22**(4): 1075-9.

13. Jones J, Pampati S, Siegler AJ. Alignment of PrEP use and sexual behavior over four months among men who have sex with men in the southern United States. *AIDS Behav* 2022; **26**(10): 3378-85.

14. Martinez JE, Jonas KJ. Pre-exposure prophylaxis sorting among men who have sex with men. *AIDS Care* 2019; **31**(3): 388-96.

15. Bavinton BR, Hammoud MA, Holt M, et al. Changes in Sexual Behaviour Following PrEP Initiation Among Australian Gay and Bisexual Men in Relationships: Results from a Prospective Observational Study. *AIDS Behav* 2021; **25**(11): 3704-11.

16. Marcus U, Hickson F, Weatherburn P, Schmidt AJ. Estimating the size of the MSM populations for 38 European countries by calculating the survey-surveillance discrepancies (SSD) between self-reported new HIV diagnoses from the European MSM internet survey (EMIS) and surveillance-reported HIV diagnoses among MSM in 2009. *BMC Public Health* 2013; **13**: 919.

17. Bierman W, Hoornenborg E, Nellen J. Nederlandse multidisciplinaire richtlijn Pre-expositie profylaxe (PrEP) ter preventie van hiv (update 2022). 2022. <https://www.soaaids.nl/files/2022-07/20220711-PrEP-richtlijn-Nederland-versie-3-update-2022.pdf>.
